# Supplementary material for: Archetypal Analysis for population genetics
Source: PLoS Comput Biol. 2022 Aug 25;18(8):e1010301. doi: 10.1371/journal.pcbi.1010301 (PMC9451066; doi:10.1371/journal.pcbi.1010301)
Supplement: S1 Text — Detailed information and additional experiments are provided. (PDF) [file pcbi.1010301.s001.pdf]

Archetypal Analysis for Population Genetics

Julia Gimbernat-Mayol[1], Albert Dominguez Mantes[2,3,4], Carlos D. Bustamante[4], Daniel Mas Montserrat[4], Alexander G. Ioannidis[4,5,*]

1 Department of Bioengineering, Faculty of Engineering, Imperial College London, London, United Kingdom
2 Brain Mind Institute, School of Life Sciences, École Polytechnique Fédérale de Lausanne, Lausanne, Switzerland
3 Institute of Bioengineering, School of Life Sciences, École Polytechnique Fédérale de Lausanne, Lausanne, Switzerland
4 Department of Biomedical Data Science, Stanford University, Stanford, California, United States of America
5 Institute for Computational and Mathematical Engineering, Stanford University, Stanford, California, United States of America

*correspondence: ioannidis@stanford.edu

S1 Text

Human bar plot labels Tables A and B display all subpopulation labels used in Figure **3b**. The details of the dataset can be found in the Datasets subsection in the Methods section.

Table A. Bar plot labels

ID	Population	ID	Population
1	Mandenka	2	Gambian in Western Division (Mandinka)
3	Mende in Sierra Leone	4	Yoruba in Ibadan (Nigeria)
5	Esan in Nigeria	6	African-Caribbean in Barbados
7	African Ancestry in SW USA	8	Bantu Herero
9	Mozabite	10	Saharawi
11	Luo	12	Bantu South Africa
13	Dinka	14	Somali
15	Masai	16	Bantu Kenya
17	Luhya in Webuye (Kenya)	18	Bantu Tswana
19	Khomani San	20	Mbuti
21	Biaka	22	San
23	Puerto Ricans in Puerto Rico	24	Colombians in Medellín (Colombia)
25	Mexican Ancestry in Los Angeles CA USA	26	Peruvian in Lima Peru
27	Maya	28	Pima
29	Zapotec	30	Quechua
31	Mixe	32	Chane
33	Mixtec	34	Piapoco
35	Surui	36	Karitiana
37	Ami	38	Southern Han Chinese
39	Miao	40	Tujia
41	Han	42	She
43	Dai	44	Dai Chinese in Xishuangbanna, China
45	Atayal	46	Han Chinese in Beijing, China
47	Igorot	48	Korean
49	Kinh in Ho Chi Minh City, Vietnamese	50	Lahu
51	Japanese in Tokyo, Japan	52	Northern Han
53	Yi	54	Naxi
55	Hezhen	56	Daur
57	Tu	58	Thai
59	Xibo	60	Mongolian
61	Oroqen	62	Cambodian
63	Ulchi	64	Burmese
65	Even	66	Yakut
67	Altaian	68	Itelman
69	Kyrgyz	70	Tubalar
71	Eskimo Chaplin	72	Eskimo Sireniki
73	Eskimo Naukan	74	Uygur
75	Mansi	76	Chukchi
77	Aleut	78	Tlingit
79	Basque	80	Czech
81	Sardinian	82	Bergamo Italian
83	French	84	British From England and Scotland
85	Northern Europeans from Utah (CEPH)	86	Albanian
87	Tuscans from Italy	88	Iberian Populations in Spain

Domestic dog breeds details. Tables C and D show the breeds of all dogs included in our study. Details about the dataset can be found above in the Datasets subsection within the Methods section.

Table B. Bar plot labels

ID	Population	ID	Population
89	Orcadian	90	Norwegian
91	Icelandic	92	Hungarian
93	Polish	94	Estonian
95	Bulgarian	96	Finnish in Finland
97	Crete	98	Greek
99	Russian	100	Samaritan
101	Adygei	102	Lezgin
103	Abkhasian	104	Saami
105	Chechen	106	North Ossetian
107	Druze	108	Yemenite Jew
109	Palestinian	110	Jordanian
111	Armenian	112	Bedouin
113	Georgian Mingrelian	114	Iraqi Jew
115	Turkish Cappadocia	116	Iranian
117	Papuan Highlands	118	Australian
119	Papuan Sepik	120	Bougainville
121	Maori	122	Hawaiian
123	Dusun	124	Gujarati Indians in Houston, Texas, USA
125	Indian Telugu in the U.K.	126	Sri Lankan Tamil in the UK
127	Brahmin	128	Yadava
129	Mala	130	Kapu
131	Punjabi in Lahore (Pakistan)	132	Madiga
133	Relli	134	Irula
135	Bengali in Bangladesh	136	Sindhi
137	Pathan	138	Kalash
139	Burusho	140	Balochi
141	Brahui	142	Khonda Dora
143	Makrani	144	Tajik
145	Kusunda	146	Hazara

Archetypal Analysis compositional plots. Figures A and B show further examples of Archetypal Analysis predictions with the dataset of dog genotypes. The figures show that, as the number of archetypes increases, more breeds are clustered in their individual archetype (e.g. A6 to A15 in the 15 archetypes plot), while the rest of breeds (the majority of the breeds) are represented as a combination of a few number of archetypes (e.g. A1 to A5 in the 15 archetypes plot).

Scalability. Figure C shows the runtime of ADMIXTURE and Archetypal Analysis when increasing the number of samples taken into account for the analysis.

Initialization effect. Figure D shows results of several runs of Archetypal Analysis on the dogs dataset using different initializations.

Relationship between AA, K-Means, and K-Medioids. We include additional plots comparing the $K = 3$ cluster centers of AA, ADMIXTURE, K-Means, and K-Medioids in Figure E and $K = 5$ in Figure F.

Explained Variance. We evaluate the quality of the methods by comparing the variance between the original genomic sequences, X and between the reconstructed sequences $\hat{X}$ (explained variance), defined as:

$$EV(X, \hat{X}) = 1 - \frac{Var(X - \hat{X})}{Var(X)} \tag{1}$$

Note that this is a similar metric as R^2:

$$R^2 = 1 - \frac{MSE(X, \hat{X})}{Var(X)} \tag{2}$$

Where $MSE(X, \hat{X})$ is the average mean squared error between X and $\hat{X}$. If $E(X) = E(\hat{X})$ then $EV = R^2$

Dog breed development. Genetic variation in dog breeds has been strongly influenced by selection for various phenotypes by humans leading to highly differentiated clusters [1].

In PCA space (Fig. 4a), the Alaskan Malamute, Siberian Husky, Greenland Sledge Dogs and the Wolf spread across the PC-1 axis, displaying the highest genetic variability. These dogs have shared ancestry [2], and they have been clustered together both in PCA and Archetypal Analysis space.

Ancient East Asian breeds such as Chow Chow, the Akita and the Shiba Inu were also found close to the Wolf in PC-1, which suggests their genetic similarity as one of the first domesticated dogs. This was also found in a recent study [3] that compared the genome sequences of Chow Chows and gray wolves to explore the development of East Asian breeds.

In the PC-2 axis, Bulldogs, Boxers and Bull Terriers are found to have the highest genetic variability. Bull Terriers were initially bred for dogfighting, after bull-baiting with Bulldogs was outlawed in the 1980s [4]. Boxers were also used for dogfighting. Boxers and all recognized Bulldogs are traditionally said to have had a common ancestor [4]. This might explain their genetic resemblance found in PCA space (Fig. 4a) and polygon compositional archetypes (Fig. 4b and 4c); however, although some breeds are commonly thought to be very ancient, previous genetic studies have suggested that the majority are modern creations. This could be what is reflected in the large cluster in PCA space. Excluding a few examples, most breeds were developed no earlier than the 19th century [4]. For example, the Poodle (Toy) and the Jack Russell Terrier were clustered together in PCA space as they only appeared in the last century, while the Norfolk (ears up) terrier became a distinct breed from the Norwich terrier (ears down) only 50 years ago. Older terrier breeds such as Glen of Imaal were first registered more than 100 years ago and are seen further up in the PC-2 axis.

There is currently no universally recognized criteria for grouping dog breeds, despite the exhaustive dog breed standards that have been defined [4]. The main regulatory bodies are the Kennel Club (KC) in the UK, the Fédération Cynologique Internationale (FCI) including 86 member countries, and the American Kennel Club (AKC). The categorizing systems of these organizations are based on function, but are each different. Both the KC and AKC recognize 7 groups while the FCI has 10 groups [4], and each recognize a different number of individual breeds. With Archetype Analysis, a genetically-driven grouping system can be established (Fig. 4b). This can result in some breeds being placed in non-traditional groupings. For example, the Miniature Schnauzer, although officially a terrier by regulatory bodies, was clustered with the

Poodle, as it a Terrier-Poodle mixture, but seems to be genetically closer to the Poodle. The groups found by Archetypal Analysis for 5 clusters are the Terrier group (A3, e.g. Scottish Terrier), the Spitz-type group (A1, e.g. Alaskan Malamute), the herding group (A5, e.g. German Shepherd), the old fighters group (A2, e.g. Bulldog) and the water hunting dogs (A4, e.g. Poodle). Other hunting dogs such as the Irish Wolfhound are found in between the herding and water hunting group. In contrast, the AKC puts the Boxer and Alaskan Malamute together as the Working group and puts the Miniature Schnauzer in the Terrier group. An advantage of an Archetypal Analysis-based clustering is the ability to represent dogs as combinations of the main groups (soft assignments), instead of as single categories (hard assignments). This can be beneficial as most dogs have been bred from multiple previous breeds and do not clearly belong to one individual grouping. For an increasing number of archetypes, breeds tend to be represented by single archetypes, showing a fine scale population structure that reflects human artificial breeding of dogs (Fig. 4b).

Table C. Domestic dog breeds details (1)

Breed	Clade	N	Breed	Clade	N
American Cocker Spaniel	Spaniel	10	American Eskimo Dog	Nordic Spitz	6
Afghan Hound	Mediterranean	10	American Hairless Terrier	American Terrier	10
Airedale Terrier	Terrier	3	Akita	Asian Spitz	10
Alaskan Malamute	Asian Spitz	10	American Staffordshire Terrier	European Mastiff	6
Anatolian Shepherd	Mediterranean	6	Australian Cattle Dog	UK Rural	10
Australian Shepherd	UK Rural	10	Australian Terrier	Terrier	10
Azawakh	Mediterranean	5	Basset Hound	Scent Hound	10
Beagle	Scent Hound	10	Bedlington Terrier	Terrier	7
Belgian Sheepdog	Continental Herder	10	Bearded Collie	UK Rural	3
Bichon Frise	Poodle	10	Bloodhound	Scent Hound	10
Belgian Malenois	Continental Herder	6	Bernese Mountain Dog	Alpine	10
Boerboel	European Mastiff	3	Border Collie	UK Rural	10
Border Terrier	Terrier	10	Borzoi	UK Rural	10
Boston Terrier	European Mastiff	10	Bouvier des Flandres	Continental Herder	8
Boxer	European Mastiff	10	Berger Picard	New World	3
Briard	Continental Herder	10	Brittany	Pointer Setter	10
Black Russian Terrier	Drover	4	Brussels Griffon	Toy Spitz	2
Basenji	-	10	Bulldog	European Mastiff	10
Bulmastiff	European Mastiff	10	Bull Terrier	European Mastiff	10
Cairn Terrier	Terrier	10	Cane Corso	European Mastiff	9
Cardigan Welsh Corgi	UK Rural	10	Curly Coated Retriever	Retriever	6
Chihuahua	American Toy	10	Japanese Chin	Asian Toy	4
Chow Chow	Asian Spitz	10	Cirneco dell'Etna	Mediterranean	5
Cavalier King Charles Spaniel	Spaniel	10	Collie	UK Rural	10
Chinook	New World	10	Coton du Tulear	Poodle	2
Cane Paratore	New World	2	Chinese Crested	American Toy	10
Dachshund	Scent Hound	10	Dalmatian	Pointer Setter	9
Great Dane	European Mastiff	10	Dogue de Bordeaux	European Mastiff	6
Scottish Deerhound	UK Rural	10	Doberman Pinscher	Drover	10
English Cocker Spaniel	Spaniel	10	English Setter	Pointer Setter	10
English Springer Spaniel	Spaniel	10	Eurasier	-	10
French Bulldog	European Mastiff	10	Flat-coated Retriever	Retriever	10
Field Spaniel	Spaniel	4	Finish Spitz	-	10
Foxhound	Scent Hound	10	Glen of Imaal Terrier	Terrier	9
Golden Retriever	Retriever	10	Gordon Setter	Pointer Setter	10
Great Pyrenees	Mediterranean	10	Greenland Sledge Dog	Asian Spitz	10
Greyhound	UK Rural	10	German Shepherd Dog	New World	10
German Shorthaired Pointer	Pointer Setter	10	Greater Swiss Mountain Dog	Alpine	6
Giant Schnauzer	Drover	10	German Wirehaired Pointer	Pointer Setter	2
Havanese	Poodle	10	Siberian Husky	Asian Spitz	10
Ibizan Hound	Mediterranean	10	Icelandic Sheepdog	Nordic Spitz	2
Peruvian Hairless dog	New World	10	Irish Terrier	Terrier	7
Irish Setter	Pointer Setter	9	Italian Greyhound	UK Rural	10
Irish Wolfhound UK	Rural	10	Irish Water Spaniel	Retriever	10
Jack Russell Terrier	Terrier	10	Keeshond	NordicSpitz	10
Kelpie	UK Rural	2	Kerry Blue Terrier	Terrier	4
Komondor	Mediterranean	2	Kuvasz	Mediterranean	10
Labrador Retriever	Retriever	10	Large Munsterlander	Pointer Setter	3
Leonberger	Mediterranean	10	Lhasa Apso	Asian Toy	10
Levriero Meridionale	Mediterranean	2	Mastino Abruzzese	Mediterranean	2
Maltese	Poodle	10	English Mastiff	European Mastiff	10
Miniature Bull Terrier	European Mastiff	10	Toy Mnachester Terrier	Pinscher	2
Miniature Pinscher	Pinscher	10	Miniature Schnauzer	Schnauzer	10

Table D. Domestic dog breeds details (2)

Breed	Clade	N	Breed	Clade	N
Neapolitan Mastiff	European Mastiff	6	Chinese Shar-pei	Asian Spitz	10
Norwegian Elkhound	Nordic Spitz	10	Shiba Inu	Asian Spitz	8
Newfoundland	Retriever	10	Shih Tzu	Asian Toy	10
Norfolk Terrier	Terrier	10	Silky Terrier	Terrier	4
Norwich Terrier	Terrier	10	Schipperke	Toy Spitz	10
Nova Scotia Duck Tolling Retriever	Retriever	10	Sloughi	Mediterranean	5
Old English Sheepdog	UK Rural	10	Spinone Italiano	Pointer Setter	2
Otter Hound	Scent Hound	9	Shetland Sheepdog	UK Rural	10
Papillon	Toy Spitz	10	Standard Schnauzer	Schnauzer	10
Parsons Russell Terrier	Terrier	2	Stafforshire Bull Terrier	European Mastiff	10
Petit Basset Greffon Vendeen	Scent Hound	10	Saint Bernard	Alpine	10
Pekingese	Asian Toy	10	Swedish Valhund	Nordic Spitz	6
Pembroke Welsh Corgi	UK Rural	10	Tibetan Mastiff	Asian Spitz	10
Pharoah Hound	Mediterranean	2	Tibetan Spaniel	Asian Toy	10
Pomeranian	Small Spitz	10	Tibetan Terrier	-	10
Poodle - Miniature	Poodle	10	Belgian Tervuren	Continental Herder	10
Poodle - Standard	Poodle	10	Toy Fox Terrier	American Terrier	4
Poodle - Toy	Poodle	10	Vizsla	Pointer Setter	7
Portuguese Water Dog	Poodle	10	Volpino Italiano	Small Spitz	4
Pug Dog	Toy Spitz	10	Weimaraner	Pointer Setter	10
Puli	Hungarian	4	Wire Fox Terrier	Terrier	10
Pumi	Hungarian	5	Whippet	UK Rural	10
Rat Terrier	American Terrier	2	Wirehaired Pointing Griffon	Pointer Setter	6
Redbone Coonhound	Scent Hound	2	West Highland White Terrier	Terrier	10
Rhodesian Ridgeback	European Mastiff	9	Xigou	Asian Spitz	5
Rottweiler	Drover	10	Xoloitzcuintle	New World	5
Saluki	Mediterranean	19	Xoloitzcuintle - Miniature	New World	5
Samoyed	-	10	Yorkshire Terrier	Terrier	10
Scottish Terrier	Terrier	10	Grey Wolf	Wolf-Jackal	7
Soft Coated Wheaten Terrier	Terrier	4	Golden Jackal	Wolf-Jackal	2

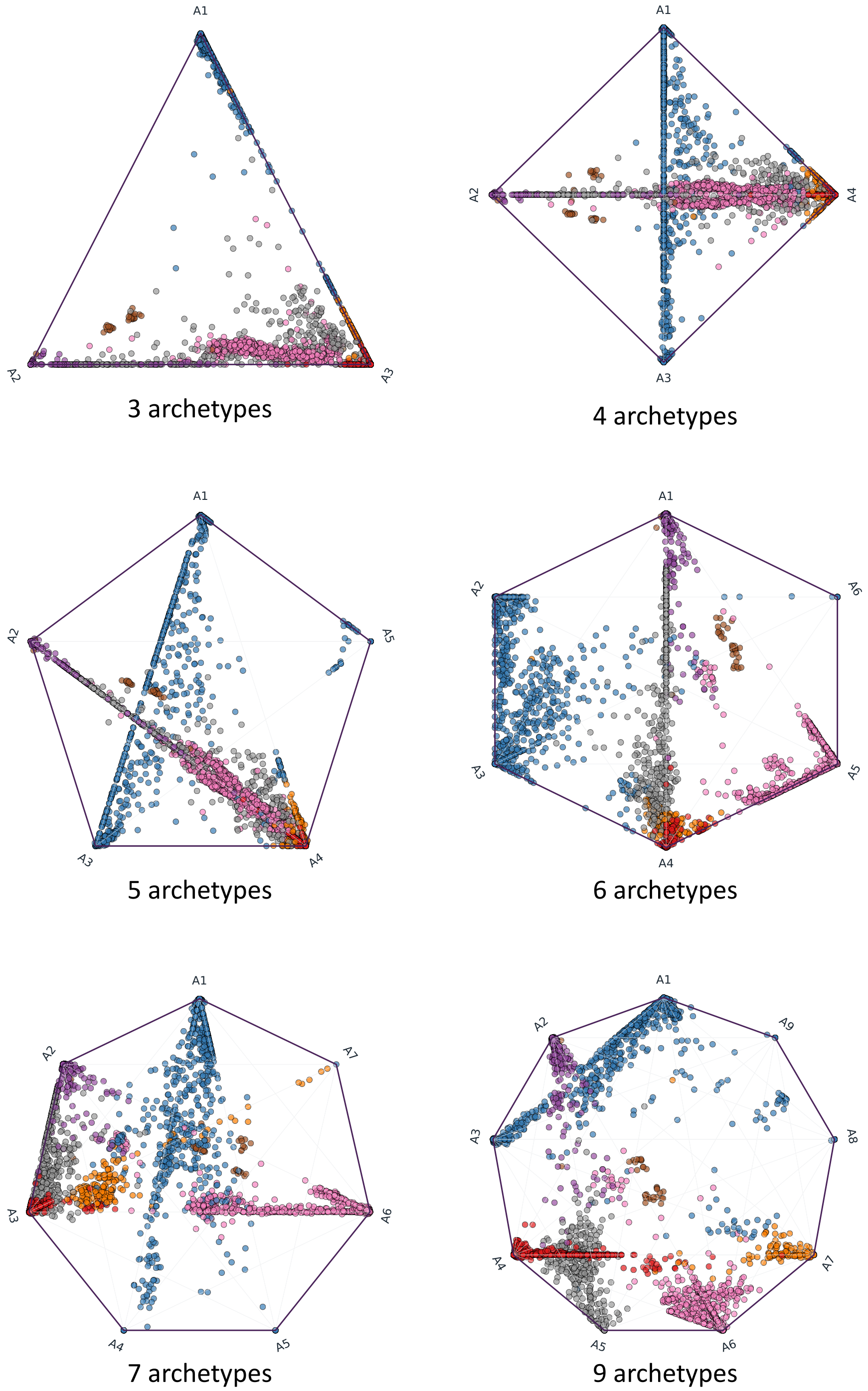

Fig A. Archetypal Analysis compositional plots for human continental populations. Archetypal Analysis polygon compositional plots of human data (3-9 archetypes, excepting the 8-archetype polygon which can be found as Fig. 2 in Section 4.1.1). The colours represent continental origins: EUR - European (red), AFR - African (blue), EAS - East Asian (purple), WAS - West Asian (orange), OCE - Oceanian (brown), SAS - South Asian (pink), AMR - American (gray).

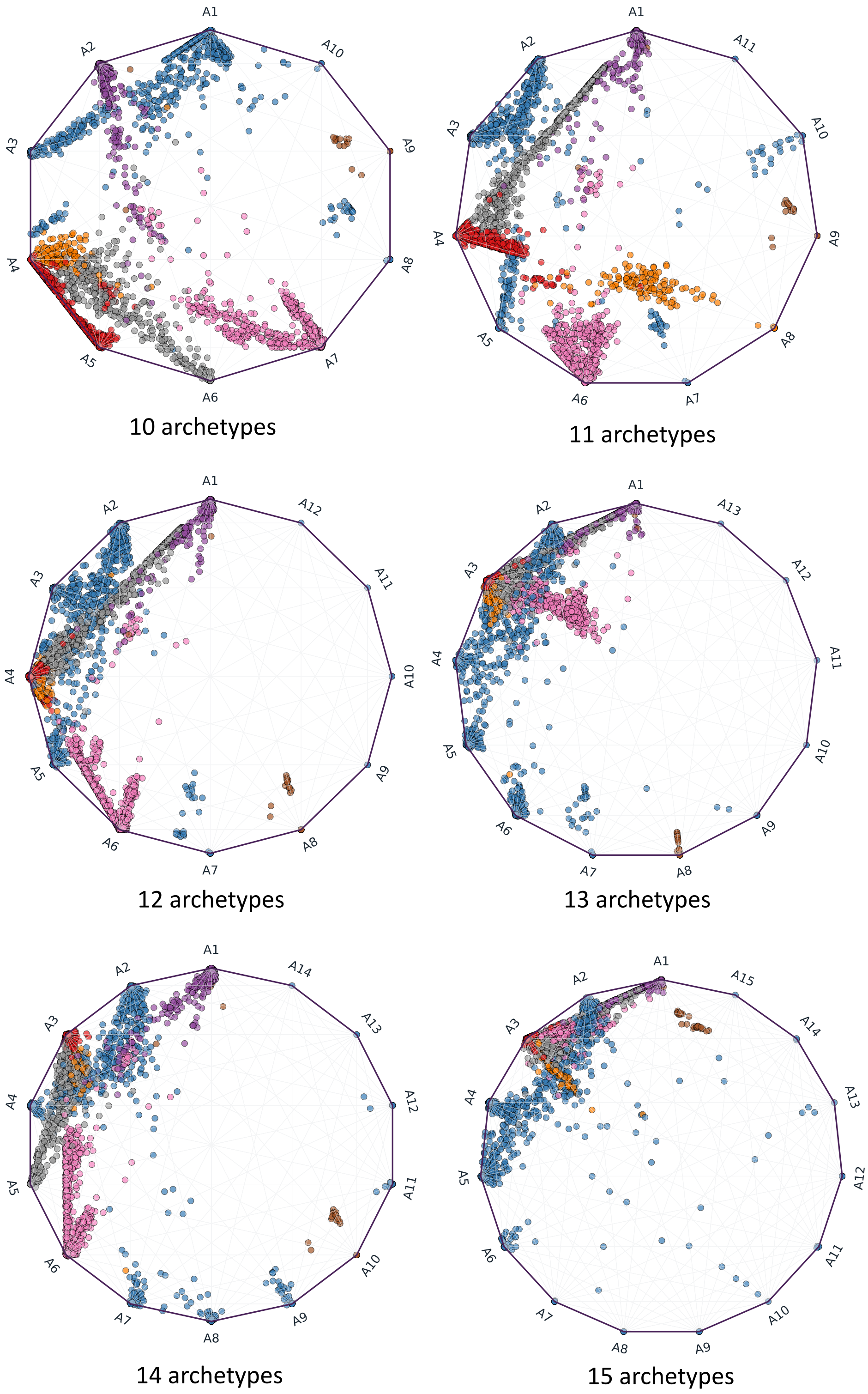

Fig B. Archetypal Analysis compositional plots for human continental populations. Archetypal Analysis polygon compositional plots of human data (10-15 archetypes). The colours represent the continental origins: EUR - European (red), AFR - African (blue), EAS - East Asian (purple), WAS - West Asian (orange), OCE - Oceanian (brown), SAS - South Asian (pink), AMR - American (gray).

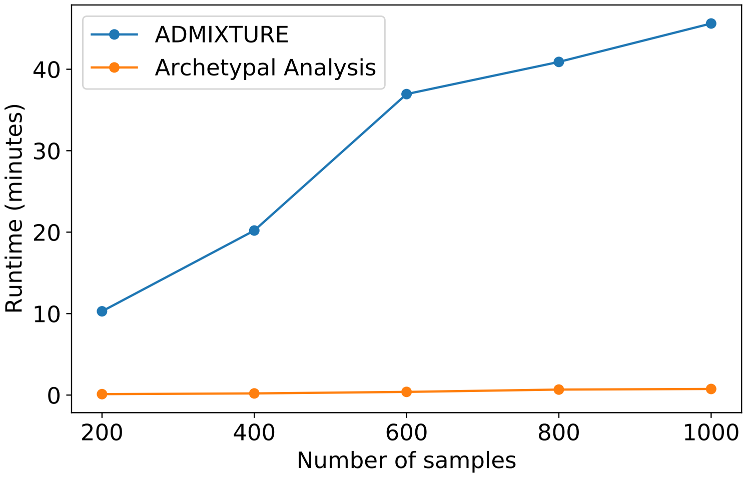

Fig C. Runtime comparison at different numbers of samples. Datasets were generated by randomly sampling individuals from the dogs dataset (150,131 SNPs) at 200, 400, 600, 800 and 1000 samples. The value of K was set to 15.

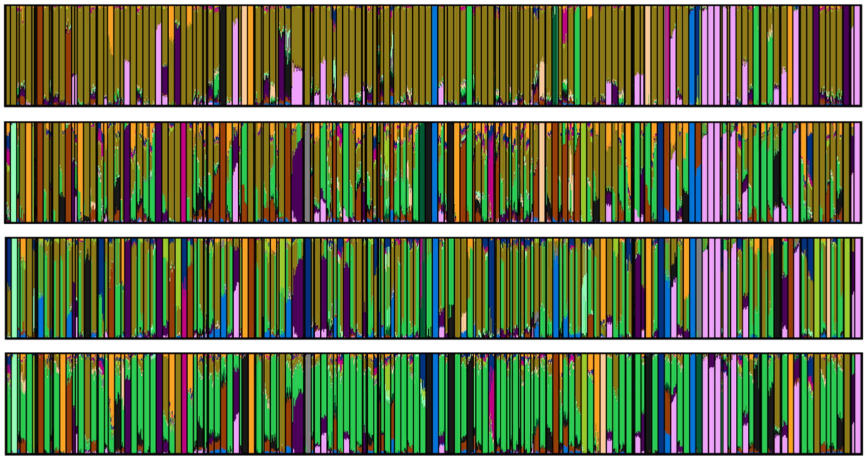

Fig D. Qualitative comparison of different runs of Archetypal Analysis ($K = 15$) on the dogs dataset using different initialization methods. The top bar plot displays results achieved using the FurthestSum initialization method, while the other 3 depict results obtained using random initializations. Variation is seen as some archetypes are split into one or more subclusters changing the bar plot.

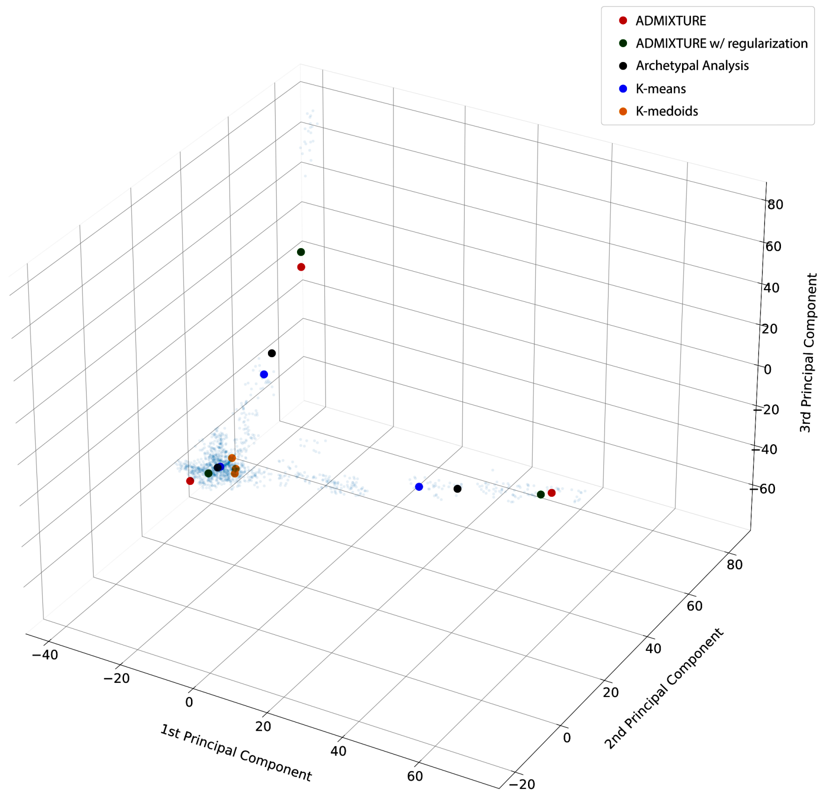

Fig E. Comparison of cluster centroids from different methods. Cluster centroids learnt by ADMIXTURE, ADMIXTURE with sparsity regularization, Archetypal Analysis, K-Means, and K-Medoids for K=3. Regularization in ADMIXTURE is introduced with lambda = 500 and epsilon = 0.1

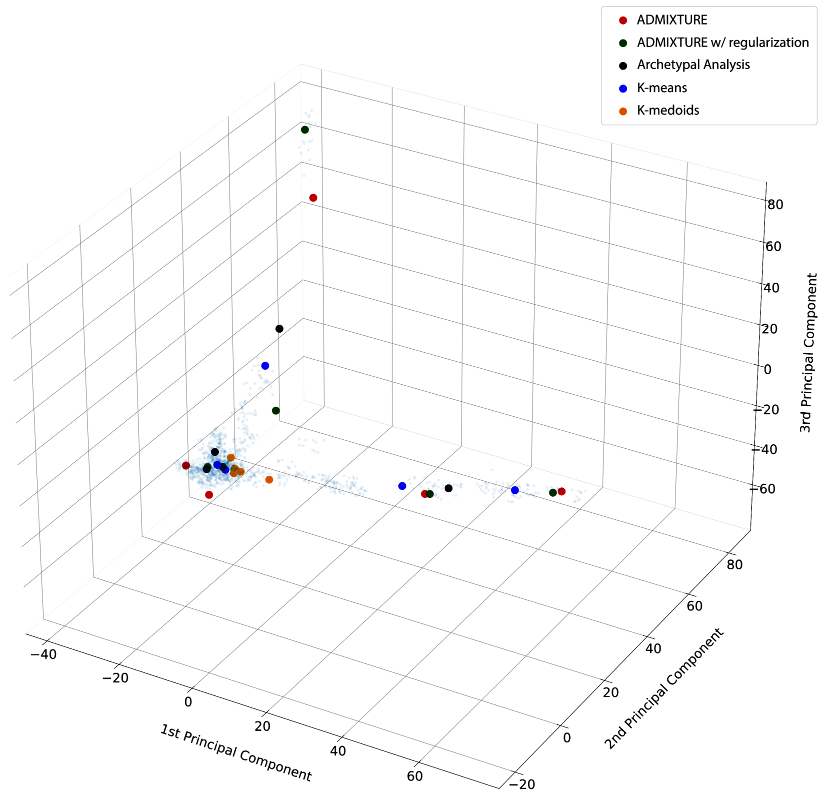

Fig F. Comparison of cluster centroids from different methods. Cluster centroids learnt by ADMIXTURE, ADMIXTURE with sparsity regularization, Archetypal Analysis, K-Means, and K-Medoids for K=5. Regularization in ADMIXTURE is introduced with lambda = 500 and epsilon = 0.1

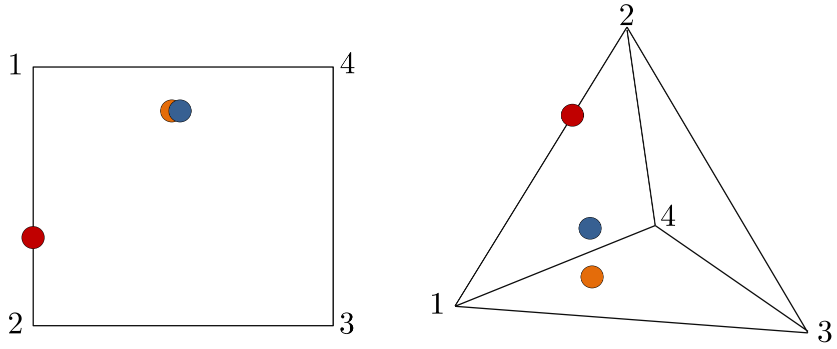

Fig G. Further intuition on interpreting compositional polygons. For four archetypes/clusters all possible proportional compositions can be represented unambiguously within the convex hull of a tetrahedron (right). A point located on a vertex (numbered) or on an edge (red dot) of the tetrahedron represents an individual's ancestry that derives from only one or two archtypes, respectively. A point falling inside a face of the tetrahedron represents an individual with ancestry deriving from three archetypes (the vertices of the face) and a point falling within the tetrahedron represents an individual with ancestry deriving from all four clusters. When collapsing the four vertices of the tetrahedron into two dimensions as the four corners of a square (polygonal) compositional plot representation, the composition of points on the edges and vertices of the tetrahedron (perimeter of the square) remain unambiguous, but multiple different points from the tetrahedron's interior and on its faces, will project to the same locations inside the square, making the composition of points plotted inside the square ambiguous. For example, the different orange and the blue points inside the 3D tetrahedron project to the same point in the 2D square. This is because there is necessarily a loss of information in such a dimensionality reduction of the compositional mapping.

References

1. Plassais J, Kim J, Davis BW, Karyadi DM, Hogan AN, Harris AC, et al. Whole genome sequencing of canids reveals genomic regions under selection and variants influencing morphology. Nature Communications. 2019;10. doi:10.1038/s41467-019-09373-w.

2. Brown SK, Darwent CM, Wictum EJ, Sacks BN. Using multiple markers to elucidate the ancient, historical and modern relationships among North American Arctic dog breeds. Heredity. 2015;115. doi:10.1038/hdy.2015.49.

3. Yang H, Wang G, Wang M, Ma Y, Yin T, Fan R, et al. The origin of chow chows in the light of the East Asian breeds. BMC Genomics. 2017;18. doi:10.1186/s12864-017-3525-9.

4. Dennnis-Bryan K, Morgan T. The Complete Dog Breed Book. Fewster H, Guerrero AG, Duffy M, Hilliard A, Berhane M, T SMT, et al., editors. Dorling Kindersley Limited; 2021.
