# Supplementary material for: Archetypal Analysis for population genetics
Source: PLoS Comput Biol. 2022 Aug 25;18(8):e1010301. doi: 10.1371/journal.pcbi.1010301 (PMC9451066; doi:10.1371/journal.pcbi.1010301)
Supplement: Attachment — Submitted filename: Archetypal Analysis for Population Genetics - response.pdf [file pcbi.1010301.s003.pdf]

<u>Archetypal Analysis for Population Genetics</u>

https://www.biorxiv.org/content/10.1101/2021.11.28.470296v1

We appreciate the support and constructive reviewer comments. We have placed our responses in-line below.

Reviewer 1:

<u>Overview</u>

Gimbernat-Mayol et al present an application of archetypal analysis (AA), an unsupervised clustering technique, to population genetics. They compare AA to ADMIXTURE, a benchmark familiar to researchers in the field. They use publicly available data on humans as well as dogs. Their code is not currently available, though they provide a link to a private github repo.

We have now set the code repo to public, and it can be accessed at https://github.com/AI-sandbox/archetypal-analysis for interested users.

The work is interesting, technically sound, and a useful contribution to the population genetics software library. Their approach is considerably faster than ADMIXTURE and has comparably useful results. The authors sufficiently discuss the technical aspects of AA as well as its relationship to other clustering approaches.

The figures in the paper will require some clarification, both in terms of how they are meant to be interpreted, and how to understand the differences between AA and ADMIXTURE. The paper would benefit from more discussion on interpretation of AA results as well as discussion of where AA and ADMIXTURE disagree on population clusters.

Specific comments are given below.

<u>Figures</u>

- The compositional plots are difficult to understand. An explanation would be useful. I understand that a point located on a vertex or an edge means that an individual's ancestry comes from one or two groups, respectively, but for some (e.g. Puerto Ricans) it is difficult to parse. It would be useful to have a figure (possibly supplementary) comparing ADMIXTURE compositional plots.

We have now included an explanatory figure of the compositional plots in the Appendix (Fig. 13), that gives further intuition on how to interpret them. A point located on a vertex represents an individual's ancestry coming from only one group, and a point located on an edge (red dot) represents an individual with ancestry that

comes from two groups: the two flanking vertices. When three ancestries are combined individuals can be represented by points lying inside a triangle. An individual with ancestry from four groups, however, must be represented as a point in three dimensions: inside a tetrahedron. When this 3d simplex is projected into only two dimensions (flattening the tetrahedron to a square), information is necessarily lost. For example, the orange and the blue dots that represent different compositions inside the tetrahedron project to the same place in the 2D square. The loss of information in this type of dimensionality reduction, means that points inside the compositional plot can be ambiguous (blue/orange dot) and could represent several different possible ancestry combinations (blue vs. orange dots) inside the original simplex.

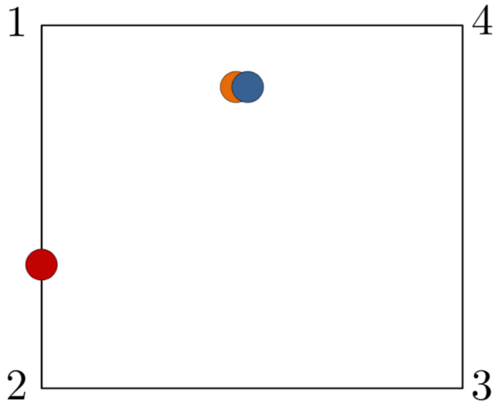

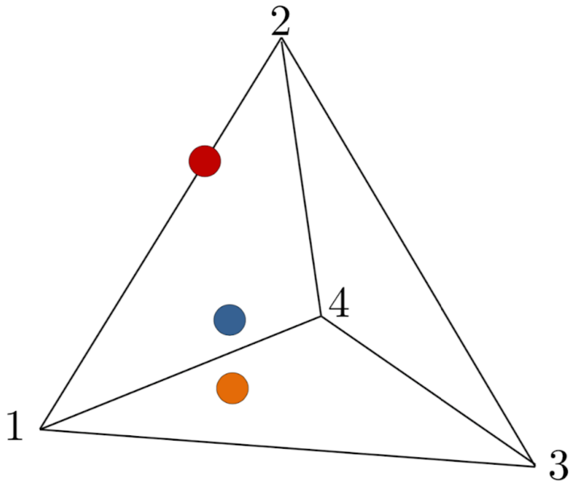

We have also included the following ADMIXTURE compositional plot of 8 ancestral clusters (Fig. 14) for comparison purposes to Fig 2 b) as suggested. This plot nicely illustrates a difference between archetypal analysis (for which archetypes represent actually realizable ancestries) and ADMIXTURE (for which cluster centers could represent SNP frequencies that could not be realized through any natural combination of the individuals in the dataset and may in fact never have existed: note that some of the vertices of the compositional plot for ADMIXTURE have no points at or near them).

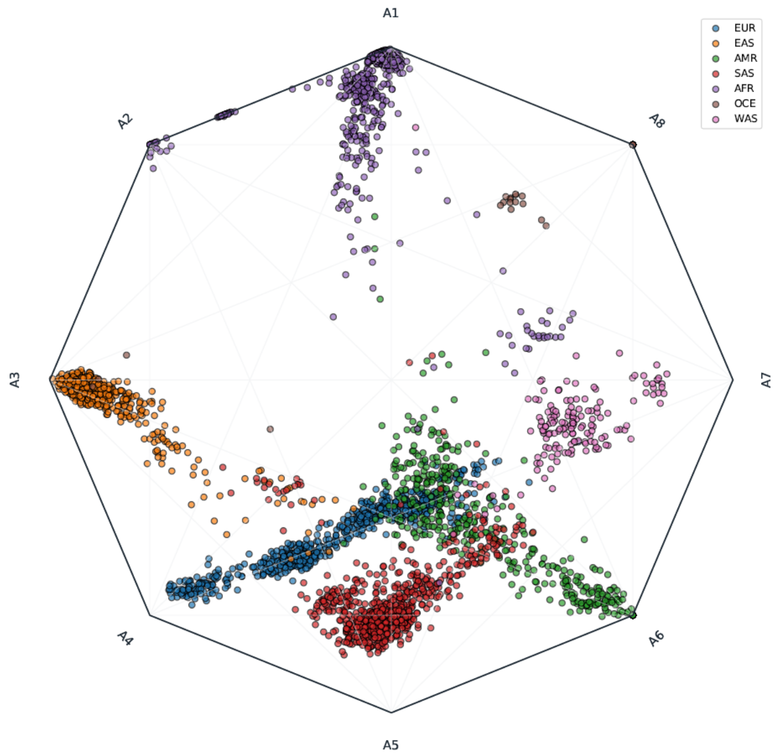

- ○ Figure 2 would be much clearer if the colours between A and B matched
- ○ There is a stray “pca_res” label in the bottom right

Colours for Figure 2 now match, and the ‘pca_res’ label has been removed. Thank you for catching this. We provide the figure here and we have updated it in the manuscript.

- Figure 3 shows some considerable differences in ancestry estimates for individuals. What do we make of this? Using ADMIXTURE, most Europeans have at least two prominent ancestral clusters, but using AA, there is mostly one ancestral group.

We have added some text to discuss this. Archetypal Analysis was able to capture the high genetic variability in African populations by identifying three ancestral clusters in this large and diverse super-population, compared to only two clusters assigned by ADMIXTURE. This had an effect on the clustering of the proportionally mostly collected under a single ancestral cluster in Archetypal Analysis but given two clusters in ADMIXTURE for the same K. (An increase in clusters in one region necessitates a loss in another for the same K.) This demonstrates how Archetypal Analysis better represents genetic variability and is less influenced (relative to ADMIXTURE) by oversampling of one less diverse population (here Europeans) relative to a more diverse one (here Africans).

- I like the dog illustrations in Figure 4, they are informative and pleasant, thank you.
    - It is not clear which groups of dots refer to which breeds in 4A. Some lines or lassos indicating groups would be useful. Increasing transparency in 4A would make the plot clearer as well.
    - There are a very large number of colours in 4A. Using shapes to reduce the number of colours would help enormously (e.g. 6 shapes would reduce it to 5 colours).
    - Are the colour schemes in B and C different from A?
    - There appear to be 8 wolf dots in 4A for 7 wolf samples listed in Table 5 – is this right? Or are these similar colours

As suggested we have included circles to indicate breed groups represented by the drawings and increased overall transparency. We have also added shapes to reduce the number of colours (4 shapes x 6 colours), and matched the colour schemes in all subfigures (A, B and C). The total number of Wolf dots (now grey crosses) was 9, because both Wolves and Golden Jackals were tagged as the Wolf Clade by Parker HG, et al., which was the source of this dataset. To aid viewer interpretation, we have now renamed this label Wolf-Jackal. See last entries of Table 5 for reference outlining Breed-Clade categories.

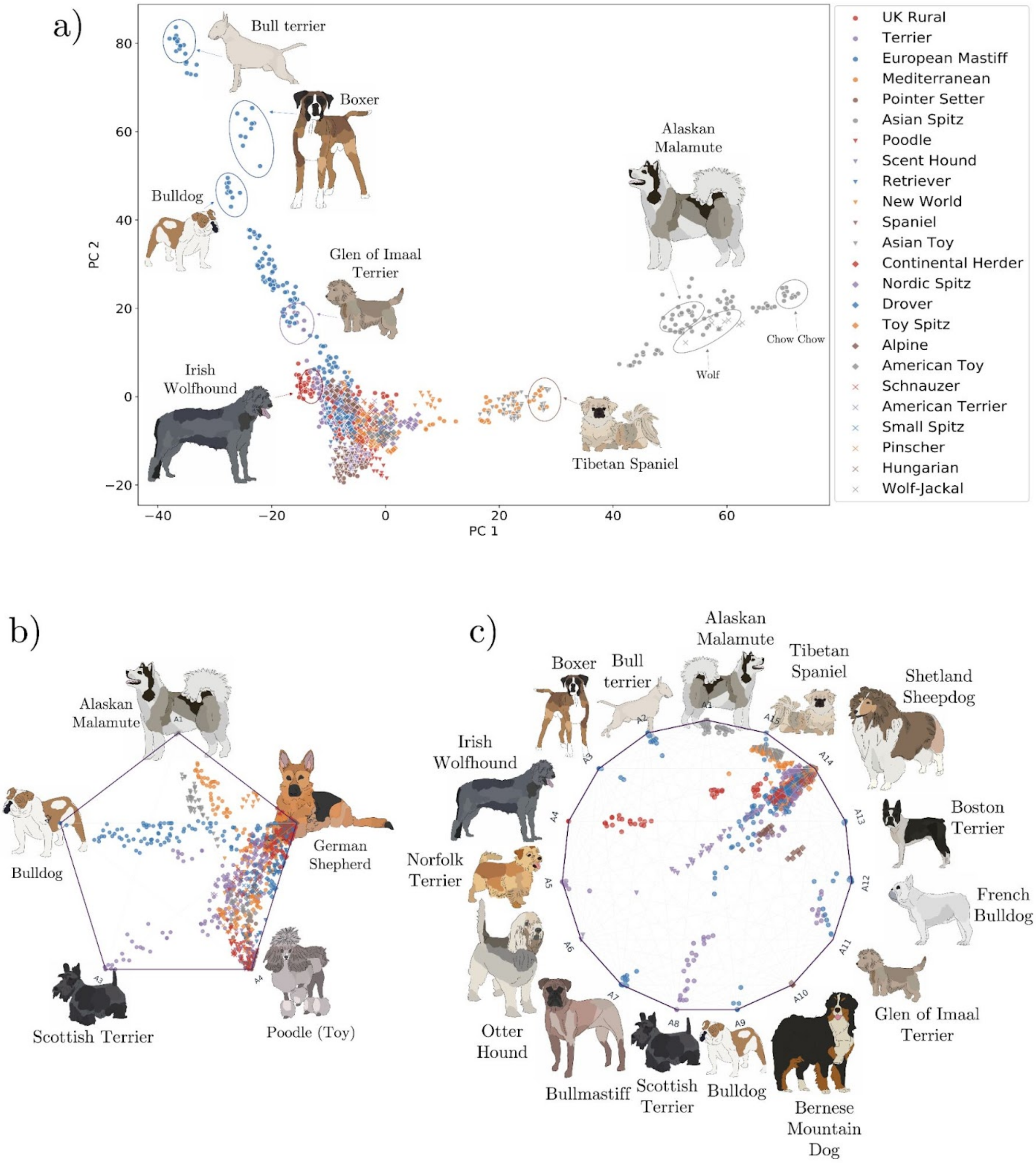

- The values of the x-axis in Figure 5B should be discrete. If you have the values for up to K=30 they should be plotted as well. Capitalization of labels between the plots is also inconsistent.

b)

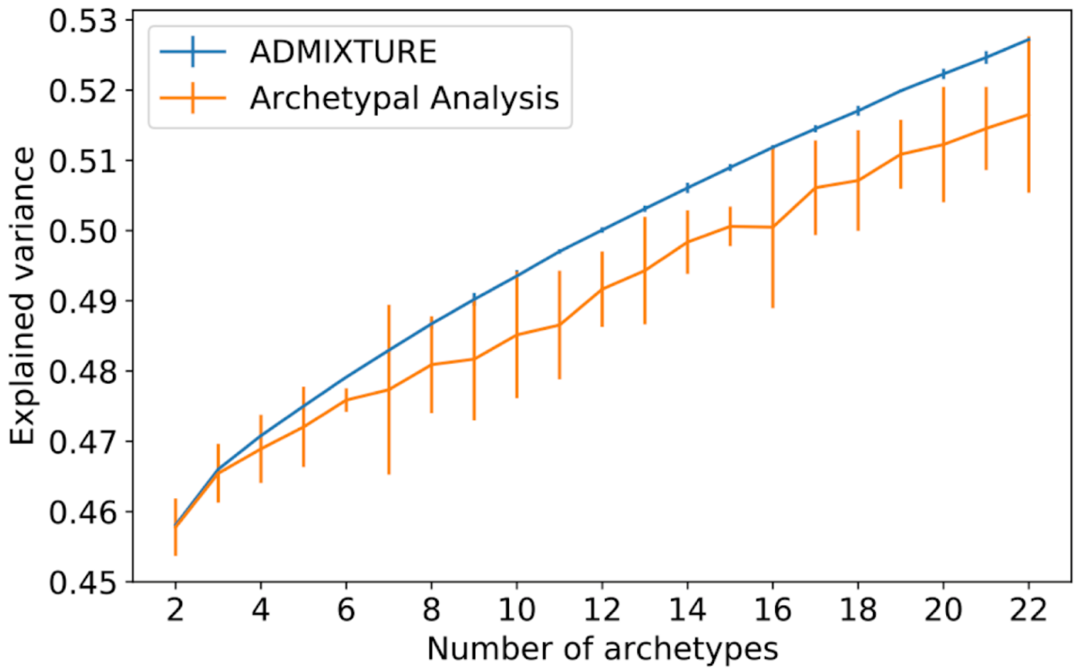

Values in the x-axis of Figure 5B have been changed to be discrete, and labels have been capitalised.

- Figure 7 has a 3-archetype figure with the points A1, A2, and A3 corresponding roughly to African, Asian, and European continental ancestries. It is interesting to me that no individuals seem to fall along the A1-A2 line segment – in my experience, a ternary plot using ADMIXTURE usually has at least a few individuals (e.g. some Central/South Americans) that fall on this axis because of their African and Indigenous ancestries. If you were to plot ADMIXTURE results the same way, does the A1-A2 line segment stay bare?

We have now additionally plotted the 3-archetype compositional plot for Admixture (Fig. 15) and show that the A1-A2 segment stays bare in this case just as for Archetypal Analysis for this dataset. This is because although some African - Indigenous admixed populations exist in the dataset (for example some Puerto Ricans and Colombians), they all have substantial European admixture as well and so lie in the interior of the triangle (gray dots) offset from the diagonal edge. The individuals closest to the A1-A2 (African - East Asian) edge are the Oceanian (brown) individuals, since they have some Austronesian (East Asian) ancestry together with a very ancient out-of-Africa component (Papuan) that is assigned partially as African at K = 3.

Comments

- The authors refer to “Native Americans” throughout the manuscript, which refers to specific populations and is not correct nomenclature for some within the group (e.g. MXL and PUR groups may have indigenous ancestry but would probably not consider themselves Native Americans or indigenous). “Americans” could be a better label (and is used in certain parts of the manuscript), or “Central/South Americans” in case they wish to distinguish from people from the USA.

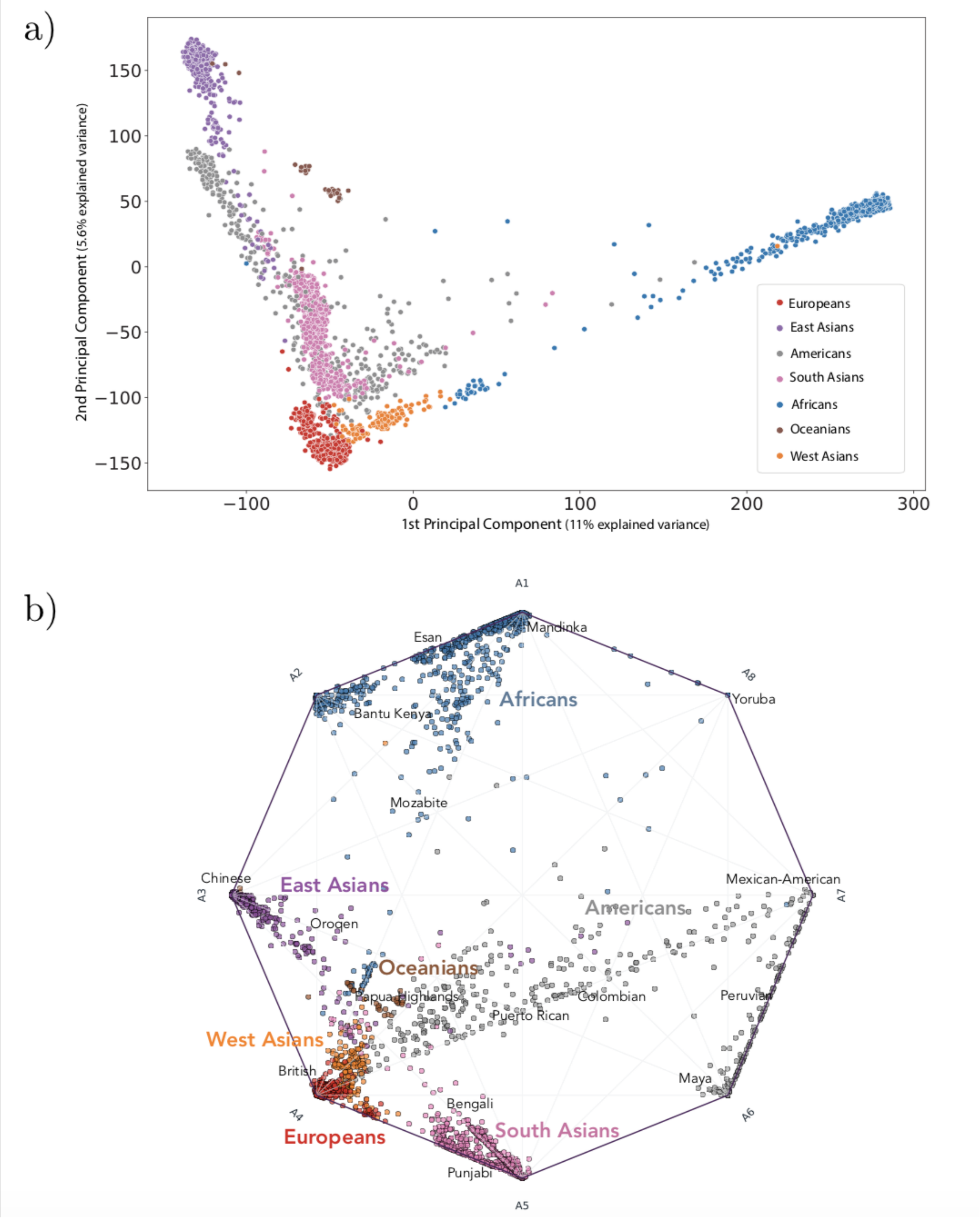

- Similar comment for the "Datasets" section, where the authors refer to 419 indigenous individuals from the Americas.

All populations previously referred to as "Native American", have now been changed to "Americans". (See example in figure above.)

- For populations from the 1000 Genomes Project, note that the project has guidelines on how to refer to populations, e.g. : 'It is important to refer to this community as "Mexican Ancestry in Los Angeles, CA, USA" … The [MXL] population should not be described as "Mexican American" out of respect for the expressed wishes of the donor community' (https://www.coriell.org/1/NHGRI/Collections/1000-Genomes-Collections/1000-Genomes-Project).

The population names for these communities have now been changed. We appreciate this point.

- Equation 6 has a typo (the 2 should be a superscript, not a subscript)

We appreciate the attention to detail. We had meant to designate the squared Frobenius norm (for matrices) (so 2 as superscript and F as subscript) and the squared L2 norm for vectors (so 2 as both subscript and superscript). We have fixed the notation throughout the paper.

- Line 113: Does the type of initialization change the results of AA?

Random initialization changes the results of AA. Quantitatively, we illustrate this in Fig. 5 through our runtime and explained variance analysis by running and averaging results over five distinct random seed runs. Ranges observed are shown in vertical bars.

- Line 146: The dog data is described as 1355 "groups". Does this mean 1355 dogs? Or have multiple dogs been pooled somehow.

The dog data includes 1355 individual dogs representing 166 dog breeds. The wording has now been changed.

- The calculation of explained variance for AA (or ADMIXTURE) is not made explicit. For PCA I understand it is the ratio of eigenvalues – is the explained variance for AA calculated similar to how linear regression uses RSS to calculate the R^2? Or is this explained variance derived from projection the matrix *Z* into PCA space?

The calculation of explained variance, EV, is EV(X, X') = 1 - Var(X - X')/Var(X) where X' is the predicted matrix and X is the ground truth matrix. Note that, under the assumption that E(X - X') = 0, this is equivalent to R2 = 1-MSE(X, X')/Var(X), where

MSE represents the mean squared error (reconstruction error), so basically our computation of EV is almost "how linear regression uses RSS to calculate the R2". As a side note, if used to evaluate PCA, this is equivalent to the explained variance in regular PCA literature (ratio of eigenvalues becomes equivalent to the RSS/MSE definition of EV). In our paper, we use EV(X, X') to evaluate our complete system (PCA+Archetypal Analysis). In pseudocode, we can consider X' = PCA_Inv(Archetypal_Analysis(PCA(X))) where PCA() and PCA_inv() are the projecting and inverse functions of PCA, and Archetypal_Analysis() a function computing the data reconstruction through the archetypal analysis matrix factorization. Note that because we use as many principal components as the number of samples, there is no explained variance lost when performing PCA (PCA reconstruction error = 0) . Therefore, if Y = PCA(X), then Var(Y) = Var(X), and EV(X,X') = EV(Y,Y'), (where Y' = Archetypal_Analysis(Y)), so the explained variance can be computed in both the PCA space (Y) and the data space (X). In order to avoid confusion on where the explained variance is applied, we have adapted the text of the section "Performance metric analysis" in the Results section, and added a small section in the supplementary material.

<u>Discussion</u>

- When discussing populations in Figure 3, it would be much clearer to use the numberings in the figure (e.g. "Bantu Herero, population 7")

Numberings from Figure 3 have been added to all populations referred to in the text as suggested.

- Line 257: The dark blue archetype is interesting when compared to ADMIXTURE's relatively uniform light blue clustering. Similarly, Line 198 highlights that AA misses Oceanian clusters. Is there any intuition as to why we see these differing clusterings?

Archetypal Analysis properly captures the wide variation within African populations, assigning more than one cluster to this diverse continent; however, this comes at the the cost (due to the fixed number of clusters K) of lacking a further separate unique cluster for Oceanians.

- Line 259: "European-like archetype components…" – the passive phrasing of this sentence is unclear. It would be easier to state, e.g., "the red bars found in European populations and African populations [6-9] suggest a history of migration and proximity".
- The phrase "European-like archetype" generally does not make much sense. If an archetype is found more commonly in Europeans, you can state, e.g., "the [colour] archetype, which is common in Europeans, is also assigned to [populations] in [some smaller frequency]…"

The wording was changed to the latter suggestion: “the red archetype, which is common in Europeans, is also assigned to…”

- Line 267: These populations are not easy to find in the figure and I’m not sure where to look. Can you zoom in or specify more concretely where to look?

Numberings from Figure 3 have been added to all populations referred to in the text (e.g. Brahui, population 141).

- How are we to interpret archetypes? For example, are Europeans considered one largely admixed group by AA whereas ADMIXTURE would consider them to be an admixture of two populations? Is there some correspondence between those two? E.g. if an individual’s AA values are A1=0.9 and A2=0.1, and ADMIXTURE presents them as a combination mostly of clusters C1 and C2 with a little C3, do we have A1»C1+C2, A2»C3?

Yes, this is generally the case, since many archetypes are represented by nearly the same point in allele frequency space as a corresponding ADMIXTURE cluster (see Figure 6), so that any bifurcation of clusters in one method vs. the other is generally limited to just that part of ancestry space (e.g. A1 often would equal C1 + C2).

- We know that *K* is to a degree arbitrary (as noted in e.g. https://www.ncbi.nlm.nih.gov/pmc/articles/PMC5068833/, https://www.ncbi.nlm.nih.gov/pmc/articles/PMC6092366/) and may not have meaningful or useful interpretations at a certain point (Figure 8 suggests very strong diminishing returns) – with the introduction of a new method, this seems useful to mention. Given the speed of AA, it will be a useful addition to the population geneticist’s toolbox, but it is evident that it will provide different results from other clustering methods and it would be useful to understand how these different methods mesh and to state explicitly where AA’s strengths and weaknesses lay.
    - More simply: what does AA tell us? What does it not tell us? What precautions should we take before making inferences?

The interpretation of dimensionality reduction results should always be treated carefully. Each technique (e.g. PCA, Archetypal Analysis, ADMIXTURE, K-Means, etc) makes different modelling assumptions, which will lead to different results. An important way to somewhat decouple real data structure from noise is to run multiple techniques with multiple hyperparameters and random seeds and observe the similarities and differences between them. Here is where the speed of the method becomes paramount: slow techniques such as FRAPPE or ADMIXTURE make it prohibitive to run them many times but the proposed AA framework allows to run the technique multiple times in a fast manner. We now discuss the feature of archetypal analysis more explicitly in the text that differentiates it most noticeably from ADMIXTURE, which is that due to its stronger constraints than ADMIXTURE, AA obtains cluster centroids that could represent real individuals, lying either on or within the set

of observations. In contrast ADMIXTURE cluster centers can represent population frequencies that have never existed in the past and also cannot be realized in the present by any combination (admixture) of populations. Thus, archetypes can be interpreted as representing actual populations, while ADMIXTURE clusters often cannot. This difference is now illustrated more prominently by the inclusion of Figure 2c.

Reviewer 2:

In this manuscript, Gimbernat-Mayol et al. propose a method for determining the ancestry composition of cohort data by using Archetypal Analysis (AA). They argue that using AA is more computationally efficient than traditionally used methods such as ADMIXTURE, and that it results in similar genetic cluster assignments.

Their computational efficiency improvement is impressive, but I think an expansion of the analyses presented in manuscript is necessary to provide the reader with a more full perspective on the benefits and drawbacks of the methodology proposed.

Expansion of the discussion as it pertains to the biological analyses would also be helpful. I detail these and other specific questions below.

Broader Comments:

1. The authors note that several programs represent competing methods that could be used instead of AA for the purposes of ancestry clustering. However in the manuscript only ADMIXTURE is benchmarked. It would be useful to see comparisons to the other two methods listed (line 8) – STRUCTURE and FRAPPE – to assist in justification of the improvement of AA compared to other existing tools. At least efficiency benchmarking would be helpful if the authors do not wish to present full results from the empirical datasets.

We have now included the suggested further benchmarking with FRAPPE. The following figure shows a runtime comparison for FRAPPE, ADMIXTURE and Archetypal Analysis for K=2 to K=30. Time is expressed in units of accumulated hours. Note that for FRAPPE we only include up to K=5 due to the severe computational limitations of Frappe (larger values of K take days to compute). We could not run STRUCTURE for any value of K due to its extreme memory usage (on a 256GB RAM node - the official software ran out of memory). However, as stated in the paper of FRAPPE and ADMIXTURE, both methods surpass STRUCTURE in speed, therefore, we can conclude that Archetypal Analysis also surpasses STRUCTURE in speed (and in memory use!)

a)

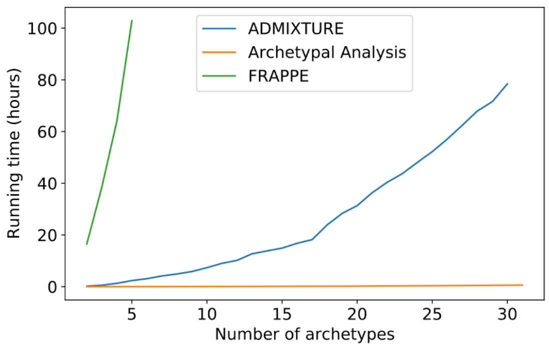

2. How are users to determine the best fit value of k in their data with AA? With ADMIXTURE, users can run a cross-validation procedure to determine the value of k that has the best predictive accuracy. These CV errors are typically plotted across increasing ks to visualize the elbow in the dataset. Such a plot would also be a helpful additional figure to show the concordance between AA and ADMIXTURE in terms of the best fit k to the data.

Figure 5b shows the explained variance for increasing K, which is inversely related to the CV error. Just as with ADMIXTURE, plots such as these can be used to determine an optimal cluster number.

3. The authors note in their introductory discussion of PCA that “interpretation can often be misleading if sampling designs are irregular” (line 26). However no discussion of the impact of sample composition for ADMIXTURE or AA is presented in the manuscript. Both will be impacted to at least some degree by sample composition, and this should at least be described for downstream users in clear terms in the manuscript, if not explored empirically. For example, sample size imbalances will impact the ordering of pulling out genetic clusters with increasing values of k. Additionally, both methods will be affected by the inclusion of related individuals in the analysis. Discussion/exploration of the impact of sample composition and recommendations for QC/data inputs by downstream users is necessary to ensure proper utilization of the method.

We now discuss the fact that AA is more robust to sample imbalance, but is still affected by it. We note that Oceania does not always get its own cluster, because it is undersampled in our dataset and that AA properly captures the diverse African populations by employing more than one cluster, as opposed to ADMIXTURE, which uses more than one cluster for the over-sampled European populations instead.

4. Some of the phrasing in the text related to the human populations should be revisited, particularly in sections prior to the Discussion.
    a. Specifically, the populations of the Americas are referred to throughout interchangeably as “Native American populations” (Figure 3) or

“indigenous individuals from the Americas” (line 144) but include many populations and individuals who would not self-identify as such, but may be better described as ‘Latinx’, ‘Latin American’, or simply ‘American.’ While there is a Native American ancestry component present in these populations of the Americas, it is incorrect to refer to many of these groups themselves as ‘Native American’ populations. The authors should revisit the phrasing surrounding such groups to be sensitive to this distinction.

Populations that were previously referred to as “Native American populations” or “indigenous individuals from the Americas” are now named as “Americans”. These labels have also been changed in the figures.

b. In a similar vein, the authors refer to individuals from distinct areas of the African continent as being part of the same population at several points in the text – i.e. “The African population displays the highest genetic variability...” (line 154). There are of course many different populations within Africa with substantial genetic and ethnolinguistic diversity across them which this phrasing currently glosses over. Referring to continental groupings instead as ‘super-population’ or even simply making the population plural (--> “The African super-population displays” or “The African populations display…”) when discussing them would ensure this comes through.

As suggested we now refer to the distinct areas of the African continent as “The African populations” in plural.

5. The Discussion currently is very thin on biological takeaways from the two empirical datasets. This would be a good place to expand on both the interpretation of the differences seen between AA and ADMIXTURE, and what can be inferred based on the patterns you observe. Cite previous research to justify that the AA determinations are logical based on what we know about the population structure and history, particularly if they differ from the determinations by ADMIXTURE.

We have inserted a discussion about the additional interpretability of AA and two new sections discussing the interpretation of AA clusters in humans and in dogs.

Other Specific Comments:

6. I did not receive any Supplementary Information. Please ensure supplementary information is included in the revision.

The supplementary figures can be found at the end of the manuscript. See supporting information section. We have also included a link to the AA code: https://github.com/AI-sandbox/archetypal-analysis

7. Author Summary – you note that AA has 'representational advantages' over ADMIXTURE. What is meant by this? This should be described in the main text if this is perceived as a primary benefit of AA over ADMIXTURE.

We discuss this at more length in the main text now and have added Figure 2c to help illustrate it (in comparison to Figure 2b). By 'representational advantages' we refer to the fact that AA obtains cluster centroids that are a convex combination of training samples, while ADMIXTURE might obtain cluster centroids outside the observed sample space. Thus, AA obtains cluster centroids that could represent real individuals, lying either on or within the set of observations. In contrast ADMIXTURE cluster centers can represent population frequencies that have never existed in the past and also cannot be realized in the present by any combination (admixture) of populations. So, archetypes can always be interpreted as representing actually achievable populations, while ADMIXTURE clusters often cannot.

8. It appears that only bi-allelic variants can be included in the AA analysis. Is this the case? Can indels be used or just SNPs? Discussion of them specific dataset filtering requirements would be useful.

We applied our analysis only to biallelic SNPs; however, archetypal analysis does not require binary input data, which we illustrate by applying it in the principal component space, so indels could be accepted as input features

.

9. Line 141 – you used a MAF cutoff of 10% here. This is extremely high. Would results be the same had you included slightly less common variants in the analysis? Justify threshold choice.

The 10% cutoff was decided in order to reduce the number of SNPs and have a more manageable dataset in order to perform experimental results and benchmarking.

In order to show that the MAF cutoff choice does not have a great impact, we have run AA 5 times on the original dogs dataset and 5 times on the filtered dogs dataset, setting the MAF cutoff to 10%. We have projected the resulting Q matrices using multidimensional scaling (MDS). In order to use MDS, we have computed the distances among them using a variant of the Jaccard similarity index, as computed in the software *pong*.

From the MDS plot we see that the slight clustering variation between runs is larger than the difference in clustering due to using (red) or not using (blue) a MAF filter.

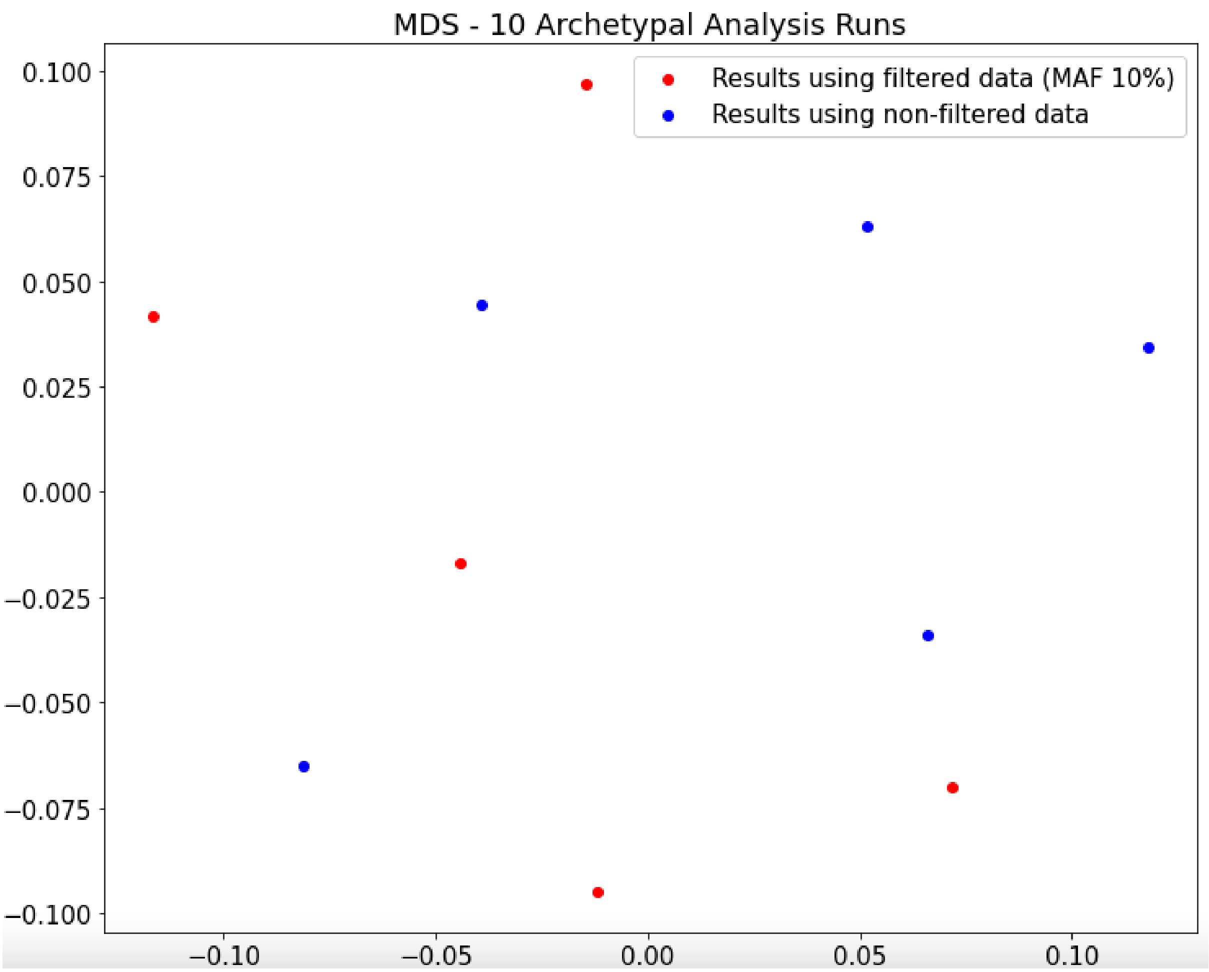

10. Line 155 – you note that "all principal components" were used but do not note how many you computed. Include the number of PCs.

In line 52 we mentioned that we are using the N-1 components as input to Archetypal Analysis. (N being the number of samples in the analysis.) We have now explicitly added this information to line 155 so as to clarify this point.

11. Figure 3 presentation – I have several suggestions to aid in the parsing of this figure.
    a. The color scheme is not consistent across panels, which would aid viewer interpretation.
    b. It is currently very difficult in panel B to determine the specific populations. The figure legend should at least point to Table 3, which contains the key to the population numbering system. A potential expansion of the x axis to allow for a more detailed population labeling may also help.

a) The colour scheme is now updated and rendered consistent across panels.

b) The figure legend now points to Table 3 and specific numbering of populations has been added in the sections where they are mentioned. A further expansion of the x-axis was not possible given the manuscript format requirements.

12. Fig 3 interpretation: There seem to be several notable differences in the ancestry component determination between ADMIXTURE and AA.
    a. Humans: The text notes the 2 vs 1 components seen in ‘Europeans’ and ‘Native Americans’, but does not discuss which orientation is more reasonable based on prior research into the population structure and history of these areas. Additionally, there’s also a distinction in Africa where ADMIXTURE appears to identify a unique San component (though it is hard to see if it is indeed the San given the population labeling system), and AA picks up a dark blue component present in many African populations that appears to be driven by the Luhya and San. Further discussion of the interpretation of differences from the two methods would be useful to include in the Discussion.

As suggested, a discussion of the interpretation of the clustering results in both humans and dogs is now included. (See response to Reviewer 1.)

13. Related, in the dog dataset, are the patterns you observe compatible with what is known about the dog phylogeny? That is, do the trends fit with the expectations from their demography?

A discussion of the results in comparison to dog demography is now included. (See below.)

Genetic variation in dog breeds has been strongly
influenced by selection for various phenotypes by humans leading to highly
differentiated clusters [18].
In PCA space (Fig. 4.5), the Alaskan Malamute, Siberian Husky, Greenland Sledge dogs and the Wolf spread across the PC-1 axis, displaying the highest genetic variability.
These dogs have shared ancestry [19], and they have been clustered together both in
PCA and Archetypal Analysis space.
Ancient East Asian breeds such as Chow Chow, the Akita and the Shiba Inu were also found close to the Wolf in PC-1, which suggests their genetic similarity as one of the first domesticated dogs. This was also found in a recent study [20] that compared the genome sequences of Chow Chows and gray wolves to explore the development of
East Asian breeds.
In the PC-2 axis, Bulldogs, Boxers and Bull Terriers are found to have the highest

genetic variability. Bull Terriers were initially bred for dogfighting, after bull-baiting with Bulldogs was outlawed in the 1980s [21]. Boxers were also used for dogfighting. Boxers and all recognized Bulldogs are said traditionally to have had a common ancestor of molossus dogs kept by the Molossians in ancient times [21]. This might explain their genetic resemblance found in PCA space (Fig. 4.5) and polygon compositions of archetypes (Fig. 4.6 and 4.7). Some breeds are commonly thought to be very ancient, but genetic evidence suggests that the majority are modern re-creations.

This could be what is reflected as the bigger cluster in PCA space (Fig. 4.5). Excluding a few examples, most breeds were developed no earlier than the 19th century [21]. For example, the Poodle (Toy) and the Jack Russell Terrier were clustered together in PCA space as they only appeared in the last 100 years, while the Norfolk (ears up) terrier became a distinct breed from the Norwich terrier (ears down) only 50 years ago. Older terrier breeds such as Glen of Imaal were first registered more than 100 years ago and are seen further up in the PC-2 axis.

There is currently no universally recognized criteria for grouping dog breeds, despite the exhaustive dog breed standards that have been defined [21]. The main regulatory bodies are the Kennel Club (KC) in the UK, the F ´ed ´eration Cynologique Internationale (FCI) including 86 member countries, and the American Kennel Club (AKC). The categorizing systems of these organizations are based on function, but are all different.

Both the KC and AKC recognize 7 groups while the FCI has 10 groups [21] and they recognize a different number of individual breeds. With Archetype Analysis, a genetically-driven grouping system can be established (Fig. 4.6). This can result in some breeds placed in unexpected groups. For example, the Miniature Schnauzer,

although officially a terrier by regulatory bodies, was clustered with the Poodle, as it a Terrier-Poodle mixture, but seems to be genetically closer to the Poodle. The groups found by Archetypal Analysis for 5 clusters are the Terrier group (A3, e.g. Scottish Terrier), the Spitz-type group (A1, e.g. Alaskan Malamute), the herding group (A5, e.g. German Shepherd), the old fighters group (A2, e.g. Bulldog) and the water hunting dogs (A4, e.g. Poodle). Other hunting dogs such as the Irish Wolfhound are somewhere in between the herding and water hunting group. In contrast, the AKC groups the

Boxer and Alaskan Malamute together as the Working group and the Miniature Schnauzer in the Terrier group. Thus, a grouping alternative based on genetic variability instead of function can explain historical relationships between dog breeds.

Another advantage of an Archetypal Analysis-based grouping system is the possibility to represent dogs as combinations of the main groups (soft assignments), instead of single categories (hard assignments). This can be beneficial as most dogs have been bred as mixtures of multiple previous breeds and do not clearly belong to one individual group. For an increasing number of archetypes, breeds tend to be represented by single archetypes, showing a growing population structure that reflects human artificial breeding of dogs (Fig. 4.7).

Previous literature suggests that Archetypal Analysis reflects a multi-objective optimization problem where biological systems adapt to optimally perform multiple tasks or condition themselves to a specific environment [22]. In particular, they find the archetype points are specialized at performing one of the tasks in an optimal manner [23]. This also seems to be the case with domestic dog breeds where selected traits that offer particular advantages to humans are highlighted as cluster representatives. For example, short-legged dogs such as Scottish Terriers are ideal for rabbit hunting [21] as their size allows them to move faster and catch their smaller preys, while long-legged dogs [21] such as the Irish Wolfhound gives hunting abilities in pursuing wolves. Consequently, the Irish Wolfhound was placed as the A4 cluster representative (Fig. 4.7), while the Scottish Terrier was placed as the A8 representative.

14. How correlated are the ancestry fractions across AA and ADMIXTURE? A quantitative comparison would complement the qualitative comparison.

The linear correlation between the results is ~0.84, while the average pairwise similarity computed using a variant of the Jaccard Index (computed using the software *pong*) is ~0.86. These figures are now included in the text.

15. How did you decide on the best fit value of k for your empirical datasets? Justify your choice of the k chosen to be presented in the primary figures (8 for humans, 15 for dogs).

Best K's are generally chosen by cross validation, and we applied the same procedure with AA for humans. This cross validation can be seen in Figure 5c. In the case of the dogs there was no clear elbow in the curve, so an intermediate value (K 15) was chosen for illustrations.

16. In studies examining admixture it is typical to run multiple ADMIXTURE runs with different seeds to confirm the results are consistent. I would suggest the authors do this, as the ancestry composition determined is a primary focus of their paper. Running 10x at different seeds for each value of k, for example, would show if there are different likely modes in the data or if the same result is always determined. It appears this was done to generate Figure 5, but that Figures 3 and 4 may be based only on one run each.

    This was done to generate Figure 5, and we show some different visualizations for below.

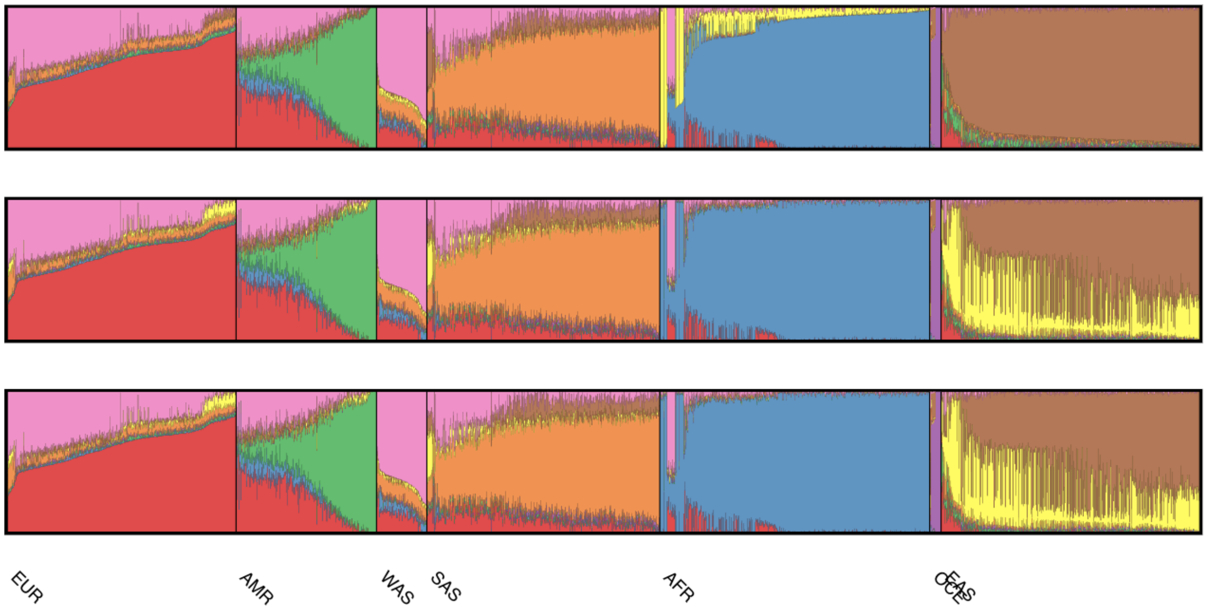

17. Fig 5 – how does runtime scale with increasing sample size?

In order to assess this, we have performed a runtime comparison between ADMIXTURE and Archetypal Analysis at different numbers of samples. Datasets were generated by randomly sampling individuals from the dogs dataset (150,131 SNPs) at 200, 400, 600, 800 and 1000 samples. The value of K was set to 15.

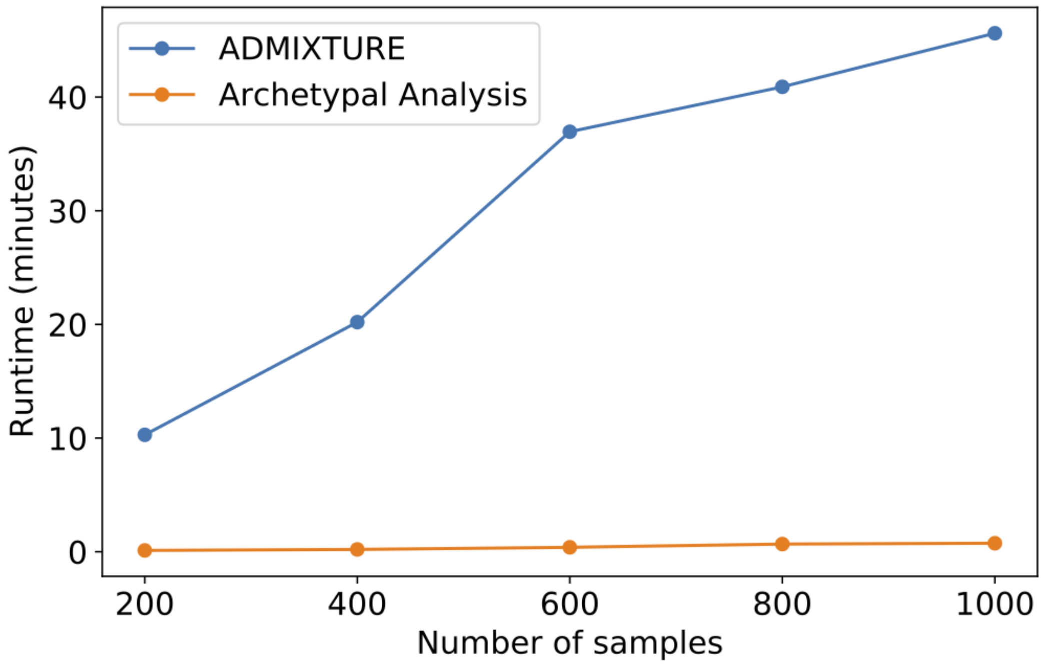

18. Line 264 – “a gradient of relatedness to Europeans.” This phrasing is a bit confusing as you don’t mean relatedness in the technical genetic meaning. I would change the wording to something like ‘European ancestry component’ or ‘cline of European admixture’ or similar.

The wording has been changed to “a cline of European admixture”.
