# Supplementary material for: Archetypal Analysis for population genetics
Source: PLoS Comput Biol. 2022 Aug 25;18(8):e1010301. doi: 10.1371/journal.pcbi.1010301 (PMC9451066; doi:10.1371/journal.pcbi.1010301)
Supplement: Attachment — Submitted filename: Archetypal Analysis for Population Genetics.docx [file pcbi.1010301.s002.docx]

**Archetypal Analysis for Population Genetics**

Overview

Gimbernat-Mayol et al present an application of archetypal analysis (AA), an unsupervised clustering technique, to population genetics. They compare AA to ADMIXTURE, a benchmark familiar to researchers in the field. They use publicly available data on humans as well as dogs. Their code is not currently available, though they provide a link to a private github repo.

The work is interesting, technically sound, and a useful contribution to the population genetics software library. Their approach is considerably faster than ADMIXTURE and has comparably useful results. The authors sufficiently discuss the technical aspects of AA as well as its relationship to other clustering approaches.

The figures in the paper will require some clarification, both in terms of how they are meant to be interpreted, and how to understand the differences between AA and ADMIXTURE. The paper would benefit from more discussion on interpretation of AA results as well as discussion of where AA and ADMIXTURE disagree on population clusters.

Specific comments are given below.

Figures

- The compositional plots are difficult to understand. An explanation would be useful. I understand that a point located on a vertex or an edge means that an individual’s ancestry comes from one or two groups, respectively, but for some (e.g. Puerto Ricans) it is difficult to parse. It would be useful to have a figure (possibly supplementary) comparing ADMIXTURE compositional plots.
- Figure 2 would be much clearer if the colours between A and B matched
  - There is a stray “pca_res” label in the bottom right
- Figure 3 shows some considerable differences in ancestry estimates for individuals. What do we make of this? Using ADMIXTURE, most Europeans have at least two prominent ancestral clusters, but using AA, there is mostly one ancestral group.
- I like the dog illustrations in Figure 4, they are informative and pleasant, thank you.
  - It is not clear which groups of dots refer to which breeds in 4A. Some lines or lassos indicating groups would be useful. Increasing transparency in 4A would make the plot clearer as well.
  - There are a very large number of colours in 4A. Using shapes to reduce the number of colours would help enormously (e.g. 6 shapes would reduce it to 5 colours).
  - Are the colour schemes in B and C different from A?
  - There appear to be 8 wolf dots in 4A for 7 wolf samples listed in Table 5 – is this right? Or are these similar colours
- The values of the x-axis in Figure 5B should be discrete. If you have the values for up to K=30 they should be plotted as well. Capitalization of labels between the plots is also inconsistent.
- Figure 7 has a 3-archetype figure with the points A1, A2, and A3 corresponding roughly to African, Asian, and European continental ancestries. It is interesting to me that no individuals seem to fall along the A1-A2 line segment – in my experience, a ternary plot using ADMIXTURE usually has at least a few individuals (e.g. some Central/South Americans) that fall on this axis because of their African and Indigenous ancestries. If you were to plot ADMIXTURE results the same way, does the A1-A2 line segment stay bare?

Comments

- The authors refer to “Native Americans” throughout the manuscript, which refers to specific populations and is not correct nomenclature for some within the group (e.g. MXL and PUR groups may have indigenous ancestry but would probably not consider themselves Native Americans or indigenous). “Americans” could be a better label (and is used in certain parts of the manuscript), or “Central/South Americans” in case they wish to distinguish from people from the USA.
  - Similar comment for the “Datasets” section, where the authors refer to 419 indigenous individuals from the Americas.
- For populations from the 1000 Genomes Project, note that the project has guidelines on how to refer to populations, e.g. : ‘It is important to refer to this community as "Mexican Ancestry in Los Angeles, CA, USA" … The [MXL] population should not be described as "Mexican American" out of respect for the expressed wishes of the donor community’ (<https://www.coriell.org/1/NHGRI/Collections/1000-Genomes-Collections/1000-Genomes-Project>).
- Equation 6 has a typo (the 2 should be a superscript, not a subscript)
- Line 113: Does the type of initialization change the results of AA?
- Line 146: The dog data is described as 1355 “groups”. Does this mean 1355 dogs? Or have multiple dogs been pooled somehow.
- The calculation of explained variance for AA (or ADMIXTURE) is not made explicit. For PCA I understand it is the ratio of eigenvalues – is the explained variance for AA calculated similar to how linear regression uses RSS to calculate the R^2^? Or is this explained variance derived from projection the matrix *Z* into PCA space?

Discussion

- When discussing populations in Figure 3, it would be much clearer to use the numberings in the figure (e.g. “Bantu Herero, population 7”)
- Line 257: The dark blue archetype is interesting when compared to ADMIXTURE’s relatively uniform light blue clustering. Similarly, Line 198 highlights that AA misses Oceanian clusters. Is there any intuition as to why we see these differing clusterings?
- Line 259: “European-like archetype components…” – the passive phrasing of this sentence is unclear. It would be easier to state, e.g., “the red bars found in European populations and African populations [6-9] suggest a history of migration and proximity”.
  - The phrase “European-like archetype” generally does not make much sense. If an archetype is found more commonly in Europeans, you can state, e.g., “the [colour] archetype, which is common in Europeans, is also assigned to [populations] in [some smaller frequency]…”
- Line 267: These populations are not easy to find in the figure and I’m not sure where to look. Can you zoom in or specify more concretely where to look?
- How are we to interpret archetypes? For example, are Europeans considered one largely admixed group by AA whereas ADMIXTURE would consider them to be an admixture of two populations? Is there some correspondence between those two? E.g. if an individual’s AA values are A1=0.9 and A2=0.1, and ADMIXTURE presents them as a combination mostly of clusters C1 and C2 with a little C3, do we have A1≈C1+C2, A2≈C3?
  - The technical discussion comparing AA to ADMIXTURE is strong, but the interpretation could be more thorough. Do the differences between ADMIXTURE groups and assigned archetypes indicate something like the age of admixture of populations?
- We know that *K* is to a degree arbitrary (as noted in e.g. <https://www.ncbi.nlm.nih.gov/pmc/articles/PMC5068833/>, <https://www.ncbi.nlm.nih.gov/pmc/articles/PMC6092366/>) and may not have meaningful or useful interpretations at a certain point (Figure 8 suggests very strong diminishing returns) – with the introduction of a new method, this seems useful to mention. Given the speed of AA, it will be a useful addition to the population geneticist’s toolbox, but it is evident that it will provide different results from other clustering methods and it would be useful to understand how these different methods mesh and to state explicitly where AA’s strengths and weaknesses lay.
  - More simply: what does AA tell us? What does it not tell us? What precautions should we take before making inferences?
